# SUCCESSFUL LEARNING EXPERIENCES OF OT AND PT STUDENTS: ENGAGING OLDER ADULTS IN THE USE OF ASSISTIVE DEVICES

**DOI:** 10.1093/geroni/igad104.2070

**Published:** 2023-12-21

**Authors:** Tracey Zeiner, Jennifer Moore, LaVona Traywick, Krista Cox, Robin McAtee

**Affiliations:** Arkansas Colleges of Health Education School of Occupational Therapy, Fort Smith, Arkansas, United States; AR Colleges of Health Education, Fort Smith, Arkansas, United States; Arkansas Colleges of Health Education, Fort Smith, Arkansas, United States; A. T. Still University, Fort Smith, Arkansas, United States; University of Arkansas for Medical Sciences, Little Rock, Arkansas, United States

## Abstract

The Arkansas Colleges of Health Education and the Arkansas Geriatric Education Collaborative collaborated on a project to use an authentic teaching/learning approach with occupational and physical therapy students about the use of assistive technologies (AT) through educating older adults. Twenty occupational and physical therapy students were mentored by faculty/staff to collaborate with community-dwelling older adults to research, develop, and disseminate 27 video vignettes shared via Facebook. The vignettes included demonstrations using AT for self-care, home management, community engagement, and leisure participation to help older adults live safely and independently. Through this authentic learning approach, the students learned about the use and benefits of AT, interprofessional practice, and the importance of collaborating with older adults. Viewers of the videos were encouraged to seek further information, providing yet another opportunity for students to acquire additional information about how to secure AT and engage with older adults. Focus groups of faculty & staff involved in the project were held to determine student learning themes arising from this project. Through these focus groups, faculty/staff expressed that students • conveyed AT recommendations without prompts for clients served in the ACHE on-campus clinic; • demonstrated a greater understanding of available non-medical AT; • revealed greater comfort levels working with older adults; • gained an appreciation and understanding of each other’s disciplines; • attained professional development goals. It was also noted that faculty involved in the project appreciated the authentic nature of the teaching approach as opposed to more traditional teaching styles such as lectures.

